# Preliminary Characterization, Antioxidant Properties and Production of Chrysolaminarin from Marine Diatom *Odontella aurita*

**DOI:** 10.3390/md12094883

**Published:** 2014-09-23

**Authors:** Song Xia, Baoyan Gao, Aifen Li, Jihai Xiong, Ziqiang Ao, Chengwu Zhang

**Affiliations:** 1 Institute of Hydrobiology, Jinan University, Guangzhou 510632, China; E-Mails: xiasongsummer212@163.com (S.X.); gaobaoyan1211@126.com (B.G.); tiger@jnu.edu.cn (A.L.); 2 Institute of Energy Research, Jiangxi Academy of Sciences, Nanchang 330096, China; E-Mails: xjh6110@vip.sina.com (J.X.); aoziqiang628@163.com (Z.A.)

**Keywords:** *Odontella aurita*, chrysolaminarin, structural characteristics, antioxidant activity, productivity

## Abstract

A new chrysolaminarin, named CL2, with a molecular mass of 7.75 kDa, was purified from the marine diatom, *Odontella aurita*, using DEAE-52 cellulose anion-exchange chromatography and Sephadex G-200 gel-filtration chromatography. The monosaccharide and structural analysis revealed that CL2 was a glucan mainly composed of glucose, which was linked by the β-d-(1→3) (main chain) and β-d-(1→6) (side chain) glycosidic bond, demonstrated by infrared spectroscopy (IR) and nuclear magnetic resonance (NMR). The antioxidant activity tests revealed that the CL2 presented stronger hydroxyl radical scavenging activity with increasing concentrations, but less was effective on reducing power analysis and scavenging 1,1-diphenyl-2-picrylhydrazyl (DPPH) radical. The influences of nitrogen concentration and light intensity on chrysolaminarin production of *O*. *aurita* were further investigated in a glass column photobioreactor, and a record high chrysolaminarin productivity of 306 mg L^−1^ day^−1^ was achieved. In conclusion, the chrysolaminarin CL2 from *O. aurita* may be explored as a natural antioxidant agent for application in aquaculture, food and pharmaceutical areas.

## 1. Introduction

Oxidative stress causes lots of damage to biological macromolecules, such as nucleic acids, proteins, lipids and carbohydrates, which may lead to the development of chronic and degenerative ailments [1]. Although a variety of synthetic chemicals, such as phenolic compounds, are found to be effective radical scavengers, they usually have side effects. Thus, many efforts have been spent on searching for compounds with antioxidant activity and low cytotoxicity from natural materials.

Recently, accumulated evidence has demonstrated that natural polysaccharides are effective antioxidants for scavenging reactive oxygen species (ROS) [2,3,4]. Marine diatoms accumulate β-d-1,3-glucans, also called chrysolaminarin, as an energy storage carbohydrate, especially upon nutrient-depletion conditions [5]. The chrysolaminarin from several diatoms have been characterized and found to be a β-1,3-glucan with a degree of polymerization (DP) in the range of 5–60 and a degree of branching (DB) of 0–0.2 at Position 6 [6]. A large number of studies indicated that β-glucans obtained from various organisms, such as plants, algae and microorganisms, are effective agents in scavenging ROS, stimulating immunity in fish, as well as treating diseases, like cancer, infection, inflammation and influenza [7,8,9,10]. However, little attention has been devoted to the production and biological activities of chrysolaminarin from marine diatoms.

The marine diatom, *Odontella aurita*, has been industrially cultured in raceways and used as a dietary supplement rich in ω-3 polyunsaturated fatty acids (PUFAs) for several years [11,12]. Some other bioactive compounds contained in this microalga, such as fucoxanthin and phytosterols, have been isolated, structurally elucidated and proved to be beneficial to human health [13,14,15]. No studies have been reported, to our knowledge, on the structural characteristics and bioactivities of chrysolaminarin in *O*. *aurita*. In this study, a new storage chrysolaminarin was purified from *O. aurita*. Its monosaccharide composition, chemical structure and antioxidant activity were characterized. The production of chrysolaminarin from *O. aurita* was also investigated in glass column photobioreactors. This research is aimed at characterizing the structure and antioxidant capacity of a new chrysolaminarin purified from the marine diatom, *O. aurita*, and evaluating its production potential in glass column photobioreactor.

## 2. Results and Discussion

### 2.1. Isolation, Purification and Characterization of Chrysolaminarin

#### 2.1.1. Purification and Homogeneity of Chrysolaminarin

**Figure 1:**
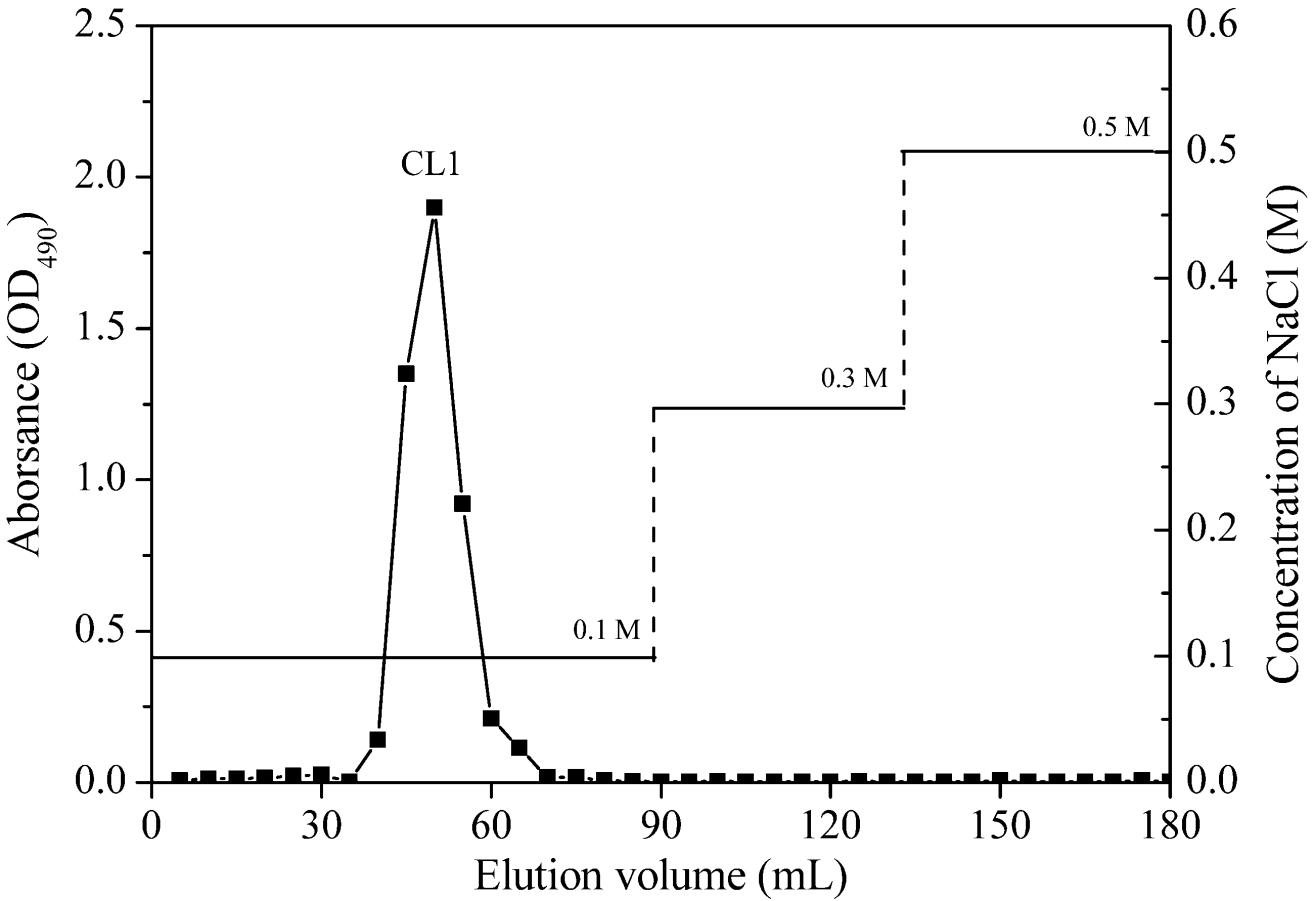
DEAE-cellulose column elution profile of crude polysaccharide from *O. aurita*.

**Figure 2:**
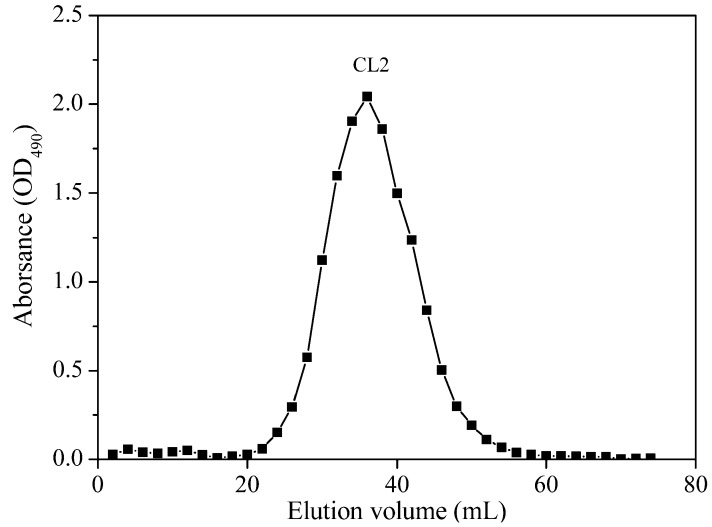
Sephadex G-200 gel-filtration chromatogram of the fraction CL1 (chrysolaminarin 1) obtained from DEAE-cellulose column elution.

The crude polysaccharide was isolated from freeze-dried *O. aurita* and then chromatographed on a DEAE-52 cellulose column (Pharmacia, Uppsala, Sweden). After being gradient eluted with an aqueous solution of NaCl (0.1, 0.3 and 0.5 M), one major peak, named CL1, was obtained from the 0.1 M NaCl eluate (Figure 1). Next, the fraction CL1 was applied to Sephadex G-200 gel-filtration column chromatography (Pharmaci, Uppsala, Sweden) for further purification, yielding a single, symmetric and sharp peak, which indicated that the obtained polysaccharide, named CL2 (chrysolaminarin 2), was homogeneous (Figure 2). The UV spectrum showed no significant absorbance at 260 nm or 280 nm, indicating that CL2 contained no protein or nucleic acid (data not shown).

#### 2.1.2. Monosaccharide Composition and Molecular Weight of CL2

Chrysolaminarins from various diatoms span a great number of different molecular weights, from ~1 to 40 kDa [6]. The average molecular weight of CL2 was found to be 7.75 kDa based on the results of gel-filtration chromatography. The monosaccharide composition of CL2 was analyzed by complete acid hydrolysis and the GC-MS analysis methods. The results shown in Table 1 indicate that the sugar components of CL2 are mainly composed of glucose (82.23%) with smaller amounts of mannose (13.27%) and traces of ribose, arabinose, xylose and galactose.

**Table 1:** Monosaccharide composition of CL2 from *O. aurita*.

Sugar Components (Total Sugar ^a^ %)					
**Glucose**	**Mannose**	**Ribose**	**Arabinose**	**Xylose**	**Galactose**
82.23	13.27	0.46	3.62	0.26	0.16

^a^ Total sugar: the sum of the six monosaccharides.

#### 2.1.3. FTIR Spectra Analysis

**Figure 3:**
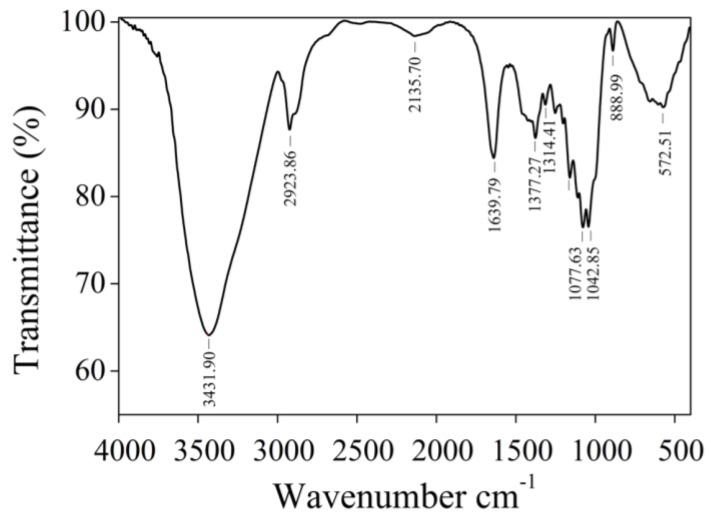
The FTIR spectra of CL2 from *O. aurita*.

As shown in Figure 3, the FTIR spectra of CL2 showed a significant, strong broad characteristic peak at around 3431 cm^−1^, corresponding to the stretching vibration of O-H groups, as well as a C-H band at 2923 cm^−1^ [16,17]. Two prominent absorption bands between 1200 and 1000 cm^−1^ are dominated by ring vibrations overlapped with C-O glycosidic band vibration [10,18,19]. The stretching peak at 1639 cm^−1^ and a weak stretching peak appeared at 1377 cm^−1^ were due to the presence of carboxyl groups [20]. The characteristic peak of β-glycosidic linkage at 889 cm^−1^ demonstrated that the obtained polysaccharide CL2 was a β-type polysaccharide [20].

#### 2.1.4. NMR Spectra Analysis

**Figure 4:**
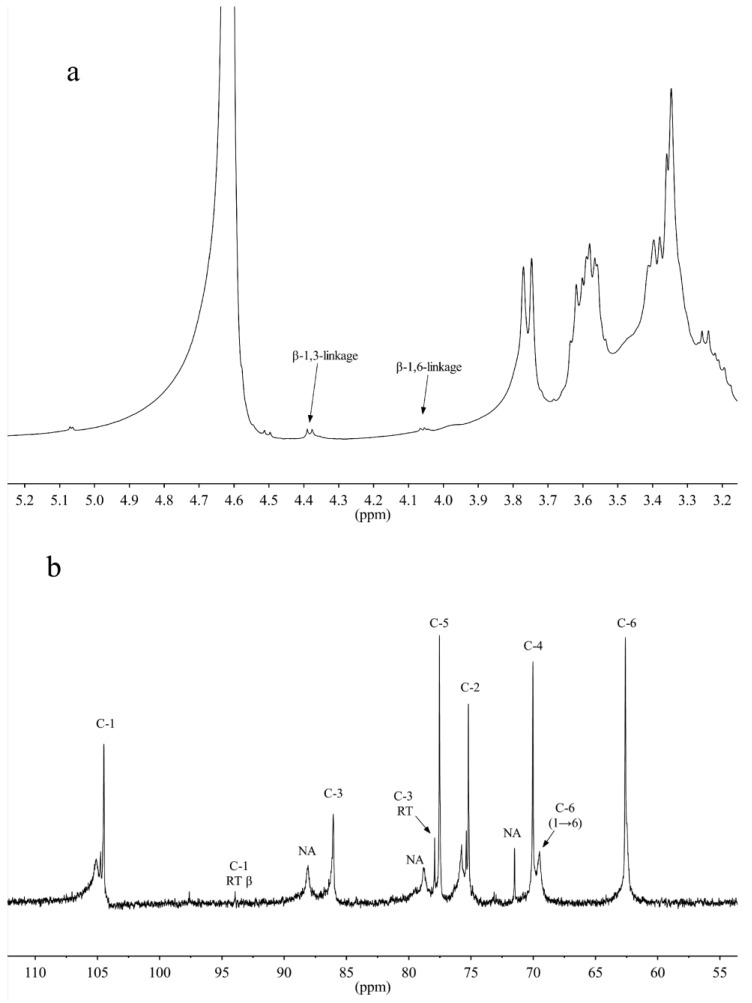
(**a**) ^1^H-NMR and (**b**) ^13^C-NMR spectrum of CL2 from *O. aurita* (NA: not assigned).

The ^1^H-NMR and ^13^C-NMR spectra of CL2 are presented in Figure 4. The anomeric proton single at δ 4.39 and 4.08 ppm in the ^1^H NMR were assigned to H-1 of the β-1,3-linkage and the β-1,6-linkage, respectively [21,22], which agree with the presence of an IR band at 889 cm^−1^. Based on the respective peak areas at 4.39 and 4.08 ppm on the ^1^H-NMR spectrum, the ratio of the β-1,3- to β-1,6-linkage was estimated to be 4:1. The ^13^C-NMR spectrum showed major signals at δ 104.2–104.6, 86.5–87.3, 78.1–78.4, 77.6–78.1, 75.1–75.4, 71.5, 69.1–70.6 and 62.3–63.1 (Figure 4b). These signals are in agreement with the results from previous analysis of microalgae *Chaetoceros muelleri* and *Pleurochrysis haptonemofera* [21,23], which indicated that the CL2 obtained from *O. aurita* has a β-d-(1→3)- (main chain) and β-d-(1→6) (branch chain)-linked glucopyranan structure. The integrated analysis of structural information demonstrated that the obtained polysaccharide CL2 from *O. aurita* is a medium molecular weight chrysolaminarin, mainly composed of a β-d-(1→3)- (main chain) and β-d-(1→6) (side chain)-linked glucose.

### 2.2. Assay for Antioxidant Activity

#### 2.2.1. Reducing Power

**Figure 5:**
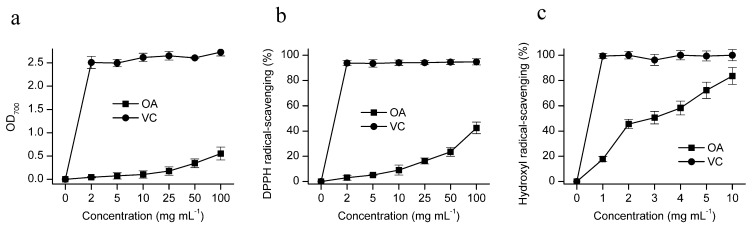
Antioxidant assays for the chrysolaminarin CL2 from *O. aurita*. (**a**) Reducing power; (**b**) scavenging of DPPH radicals; (**c**) scavenging of hydroxyl radicals. Values are the means ± SD (*n* = 3). When error bars cannot be seen, the error is less than the size of the symbol.

For the assessment of the reducing power, the Fe^3+^–Fe^2+^ transformation of CL2 was investigated using the potassium ferricyanide reduction method (Figure 5a). The results revealed that the reducing power of CL2 was weak. At 2 mg mL^−1^, CL2 showed a reducing power of 0.046 ± 0.015 abs, which gradually increased to 0.554 ± 0.139 abs at 100 mg mL^−1^; whereas the reducing power of ascorbic acid (VC) reached a plateau of 2.508 ± 0.13 abs at 2 mg mL^−1^. Kozarski *et al.* [24] declared that starch exhibited no reducing power in their study. Lo *et al.* [25] reported a weak relationship between reducing power and monosaccharide composition, and they also found that the reducing power of polysaccharides was much lower than that of ascorbic acid. Kanmani *et al.* [26] found that the exopolysaccharide from *Streptococcus phocae* exhibited a rather weak reducing power (0.2 abs at 2 mg mL^−1^), which was much lower than that of ascorbic acid (reaching its plateau of 2.5 abs at 0.4 mg mL^−1^).

#### 2.2.2. DPPH Radical Scavenging Activity

The DPPH free radical has been widely used as a tool to evaluate the antioxidant activity. The results shown in Figure 5b indicated that the scavenging activity of CL2 increased slowly with the increase of dosage in a concentration-dependent manner. When the concentration of CL2 was at 100 mg mL^−1^, its scavenging activity reached 42.455% ± 4.671%. Ascorbic acid expressed a much higher scavenging ability and reached a plateau of 93.774% ± 2.089% at 2 mg mL^−1^. The structural characteristics of polysaccharide, such as molecular weight, monosaccharide composition, availability of hydroxyl group and conformation of side chains, were reported to be responsible for the scavenging ability of polysaccharide [2,25,27].

#### 2.2.3. Hydroxyl Radical Scavenging Activity

Hydroxyl radical is believed to be the most harmful free radical in the reactive oxygen species, as it could induce severe damage to adjacent biomolecules [28]. The hydroxyl radical scavenging activity of CL2 is shown in Figure 5c. The CL2 exhibited high scavenging activity on hydroxyl radical, and the scavenging effect of polysaccharide enhanced with increasing dosage. At 1 mg mL^−1^, CL2 showed hydroxyl radical scavenging activity of 17.72% ± 1.98%, which gradually increased to 83.54% ± 6.71% at 10 mg mL^−1^. However, the scavenging activity of CL2 was much lower than that of ascorbic acid, as it reached a plateau of 99.38% ± 2.089% at 1 mg mL^−1^. The potential antioxidant activity of chrysolaminarin from *O. aurita* may lead to the development of a novel natural antioxidant agent. The results were in accord with Kanmani *et al.* [26], who found that the purified exopolysaccharide exhibited low reducing power, but relatively strong hydroxyl radical scavenging activity; at 1.2 mg mL^−1^, the hydroxyl radical scavenging activity of purified exopolysaccharide reached almost 20%, which increased to 40%–45% at 2.4 mg mL^−1^. In all experiments, the control ascorbic acid showed much better antioxidant activity than exopolysaccharide produced from *S*. *phocae*.

There are a number of reports on the evaluation of antioxidant activity in diatoms (*Phaeodactylum* *tricornutum* and *Chaetoceros calcitrans*) or other microalgae (*Botryococcus braunii*, *Porphyridium cruentum* and *Scenedesmus obliquus*) [29,30]. These studies concluded that several microalgal genera contain potent antioxidants. However, the experiments were carried out with lipophilic and hydrophilic extracts, not a purified compound. Kanmani *et al.* [26] also found that crude exopolysaccharide extract showed higher reducing power and hydroxyl radical scavenging activity than purified exopolysaccharide, which may be due to the antioxidant components, such as proteins, amino acids, organic acids and other microelements, in crude exopolysaccharide.

### 2.3. Production of Chrysolaminarin from O. aurita in a Column Photobioreactor

**Figure 6:**
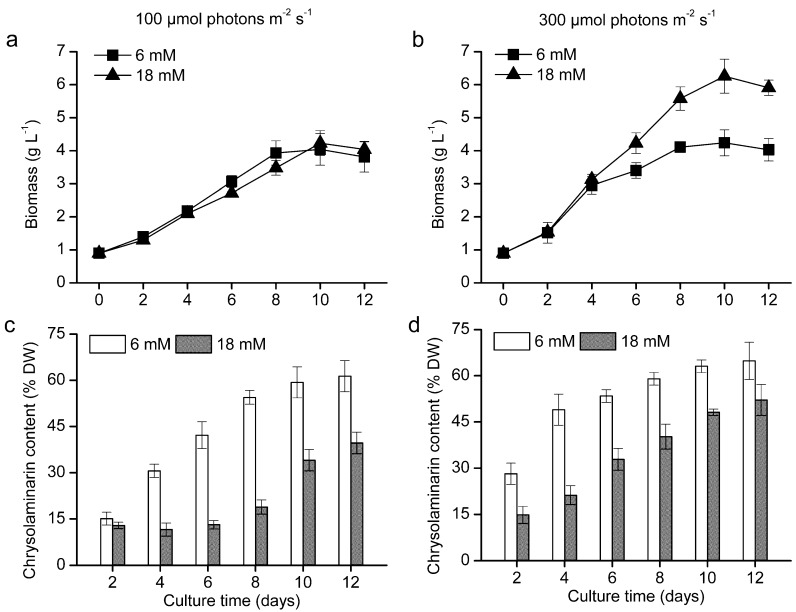
The biomass (**a**,**b**) and chrysolaminarin content (**c**,**d**) of *O. aurita* cultivated in the column photobioreactor under 100 (**a**,**c**) and 300 (**b**,**d**) μmol photons m^−2^ s^−1^ with a replete (18 mM) and deficient (6 mM) nitrate supply. Values are the means ± SD (*n* = 3). When error bars cannot be seen, the error is less than the size of the symbol.

To investigate the accumulation pattern of chrysolaminarin in *O. aurita*, the biomass concentration and chrysolaminarin content of *O. aurita* cultivated in the glass column photobioreactor were studied (Figure 6). The biomass concentration of *O. aurita* had been illustrated in our previous paper [15], which concluded that high light intensity (300 μmol photons m^−2^ s^−1^) and nitrogen-replete condition (18 mM) were favorable to obtain high biomass accumulation, and the maximum biomass concentration of 6.36 g L^−1^ was achieved on Day 10 (Figure 6b). Under low light, the chrysolaminarin content in the low nitrogen cultures increased from 15.09% of dry weight (DW) to 61.34% DW during a 12-day cultivation. In the high nitrogen cultures, chrysolaminarin content remained stable in the first eight days and then gradually increased to 39.67% DW at the end of the culture period (Figure 6c). Under high light, the changes in chrysolaminarin content followed the similar trends of their counterparts under low light with the difference being that the chrysolaminarin accumulated earlier. A maximum chrysolaminarin content of 64.86% DW was obtained in the low nitrogen cultures (Figure 6d). These results indicated that chrysolaminarin acts as storage products in *O. aurita*, especially in nitrogen-depleted condition. Myklestad [31] also found that the chrysolaminarin contents of marine diatoms *Chaetoceros affinis* and *Skeletonema costatum* were usually low in the exponential phase of growth, but they increased very rapidly when nutrients were exhausted in the stationary phase. The light intensity also affects the content of glucan; at nutrient saturation, high light led to the higher level of chrysolaminarin, which was consistent with the results in this study.

However, nutrient depletion also limited the growth of *O. aurita*, leading to the decline of biomass concentration (Figure 6a,b). Chrysolaminarin productivity, as a combined effect of biomass concentration and chrysolaminarin content, was a more suitable evaluation index for chrysolaminarin production. The chrysolaminarin volumetric productivity of *O. aurita* cultivated under different conditions was compared (Table 2). High light (HL) was demonstrated to be beneficial for maximizing chrysolaminarin productivity, as the biomass concentration and chrysolaminarin content of *O. aurita* under HL were significantly higher than their counterparts under low light (LL). Under HL, because of the significant enhancement in biomass concentration, the obtained chrysolaminarin productivity in high nitrogen (HN) was 14.18% higher than that in low nitrogen (LN), resulting in a record high chrysolaminarin productivity of 306 mg L^−1^ day^−1^. The results indicated that the microalga, *O. aurita*, may be a promising natural source for the production of antioxidative chrysolaminarin.

**Table 2:** The chrysolaminarin volumetric productivity of *O. aurita* cultivated under different conditions in a column photobioreactor at Day 10. Data represent the mean of three replicates.

Culture Condition ^a^	Biomass Concentration (g L^−1^)	Chrysolaminarin Content (% Dry Weight)	Chrysolaminarin Productivity (mg L^−1^ Day^−1^)
LL + LN	4.04	59.33	240
LL + HN	4.23	34.05	144
HL + LN	4.24	63.11	268
HL + HN	6.36	48.16	306

^a^ LL: low light (100 μmol photons m^−2^ s^−1^); HL: high light (300 μmol photons m^−2^ s^−1^); LN: low nitrogen (6 mM); HN: high nitrogen (18 mM).

## 3. Experimental Section

### 3.1. Organism and Culture Conditions

The diatom, *Odontella aurita* K-1251, was obtained from the Scandinavian Culture Collection of Algae and Protozoa (SCCAP) at the University of Copenhagen (Copenhagen, Denmark) and deposited in our laboratory with modified L1 medium. The influences of nitrogen concentration and light intensity on chrysolaminarin accumulation were conducted with column photobioreactors (60 cm length, 3 cm diameter). Cultures were aerated with air supplemented with 1% CO_2_ through a hollow glass rod and maintained at 25 ± 2 °C in an air-conditioned room. The growth medium recipe and cultural systems have been illustrated in our previous papers [5,15]. Two light intensities (100 and 300 μmol photons m^−2^ s^−1^) and two nitrogen concentrations (6 and 18 mM) were designed for investigating the accumulation pattern of chrysolaminarin in *O. aurita*. The paste of algal cells collected by centrifugation was inoculated into different treatments at roughly the same starting cell concentration based on the optical density at a 750-nm wavelength. The cultures were harvested every two days, and the corresponding biomass concentration and chrysolaminarin content were determined.

### 3.2. Biomass Measurement

Briefly, 10-mL cultures were filtered onto a pre-weighed GF/B filter paper and dried at 105 °C. The biomass concentration was determined by the difference in weight [15].

### 3.3. Determination of Chrysolaminarin Content

Chrysolaminarin was extracted from *O. aurita* according to Granum and Myklestad [32] with minor modification. Briefly, freeze-dried algal powder (50 mg) was extracted with 5 mL of 50 mM sulfuric acid at 60 °C for 30 min. The extract was assayed quantitatively for chrysolaminarin content using the phenol-sulfuric acid method [33]. Briefly, 1 mL of chrysolaminarin extract was mixed with 0.5 mL 6% (w/v) phenol solution and 5 mL concentrated sulfuric acid. After standing for 30 min, the absorbance of the mixture at 490 nm was measured, and the chrysolaminarin content was determined by comparison to a calibration curve prepared with glucose.

### 3.4. Preparation of Chrysolaminarin

**Figure 7:**
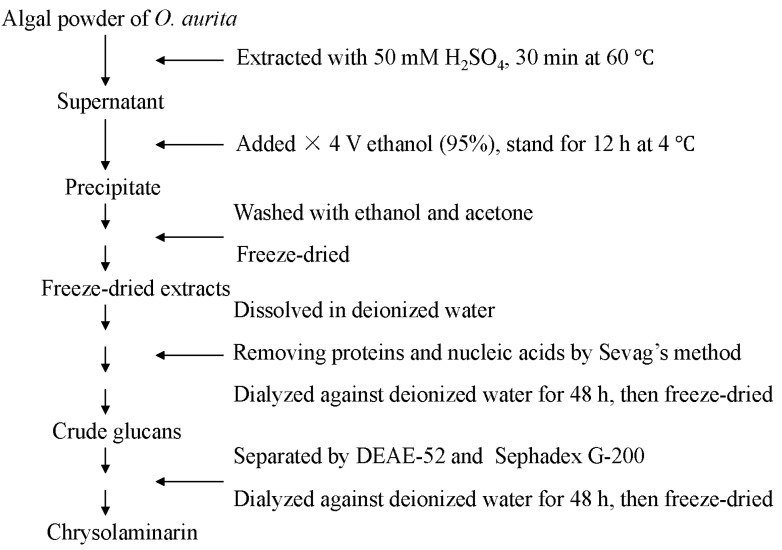
Isolation and purification procedure of chrysolaminarin from *O. aurita*.

The isolation and purification procedure of chrysolaminarin from *O. aurita* was performed according to the flowchart shown in Figure 7. Freeze-dried microalgal powder (10 g) was extracted twice with 500 mL sulfuric acid (50 mM) in a 60 °C water bath for 30 min. The supernatants were collected by centrifugation and precipitated with four volumes of 95% ethanol at 4 °C. The precipitate was recovered by centrifugation (5000 rpm, 5 min) and washed twice with ethanol and acetone, then freeze-dried. The freeze-dried extracts were dissolved in deionized water, and the proteins were removed by the mixture of chloroform: *n*-butyl alcohol (4:1, v/v) according to the Sevag method [34]. The deproteinized solution was dialyzed against deionized water for 48 h, and the crude chrysolaminarin was obtained under freeze drying. Then, 3 mL of crude chrysolaminarin solution (10 g L^−1^) were applied to a DEAE-52 cellulose chromatography column (2 × 30 cm) and gradient eluted with sodium chloride (0.1 M, 0.3 M and 0.5 M). Each 5 mL of eluate was collected at a flow rate of 0.5 mL/min and monitored for the presence of polysaccharides using the phenol-sulfuric acid method [33]. The collected fraction (named CL1) was dialyzed, concentrated and loaded on a Sephadex G-200 column (Pharmacia, Uppsala, Sweden) with 0.1 M sodium chloride as the mobile phase. Each 2 mL of eluate was collected at a flow rate of 0.2 mL/min. The corresponding chrysolaminarin fraction was collected, dialyzed against distilled water for 48 h and concentrated with air flow. Freeze drying of the resulting solution obtained the chrysolaminarin as a faintly white powder (named CL2) and gave the chrysolaminarin in a 10% yield, which was used for gas chromatography-mass spectrometry (GC-MS), infrared spectroscopy (IR) and nuclear magnetic resonance spectroscopy (NMR).

### 3.5. Structural Analysis

#### 3.5.1. Molecular Weight

The average molecular weight of chrysolaminarin CL2 was determined by comparison to a calibration curve prepared with the T-series Dextran standards (Sigma-Aldrich, ST, Louis, MO, USA) as molecular mass markers using gel-filtration chromatography (GPC) [35].

#### 3.5.2. Monosaccharide Composition

GC-MS (TRACE, Thermo Finnigan, Waltham, MA, USA) was used for analysis of the monosaccharide components. The chrysolaminarin, CL2 (10 mg), was hydrolyzed in 10 mL 3 M trifluoroacetic acid (TFA) at 105 °C for 6 h. The product was reduced with NaBH_4_ for 2 h at room temperature, acetic anhydride at 100 °C for 2 h and then analyzed by gas chromatography at a temperature program of 150–220 °C with a rate of 4 °C/min using N_2_ as the carrier. A standard curve was set up with standard monosaccharides derivatized and measured under the same procedure [10].

#### 3.5.3. IR Spectroscopy

The chrysolaminarin, CL2, was ground with dry KBr powder and pressed for Fourier transform infrared (FTIR) measurement using an EQUINOX55 spectrometer (Bruker, Bremen, Germany) at the frequency range of 4000–400 cm^−1^.

#### 3.5.4. NMR Analysis

Dried chrysolaminarin CL2 (15 mg) was dissolved in D_2_O for NMR measurements. NMR spectra were obtained on a Bruker AVANCE III 500 spectrometer (Bruker Biospin, Rheinstetten, Germany) and recorded at 500.26 MHz for ^1^H and 125.8 for ^13^C nuclei, using a 5-mm broadband probe head. Spectra were obtained at 298 K in D_2_O, with DSS as the internal reference standard. The signals were assigned regarding Størseth *et al.* [6].

### 3.6. Antioxidant Activity Assessment

#### 3.6.1. Reducing Power

The reducing power of chrysolaminarin CL2 was determined based on Deng *et al.* [10] with minor modification. Briefly, 1 mL of chrysolaminarin CL2 solution was mixed with 0.2 mL 2 M sodium phosphate buffer (pH 6.6) and 0.5 mL 1% (w/v) aqueous potassium ferricyanide. The mixture was incubated at 50 °C for 20 min in a water bath. Then, 2.5 mL 10% (w/v) of trichloroacetic acid were added to the mixture. The resultant mixture was centrifuged at 3500 rpm for 10 min. Two milliliters of the supernatant were diluted with 3 mL distilled water and then mixed with 0.5 mL 0.3% (w/v) ferric chloride. The absorbance was measured at 700 nm against distilled water. The increase in absorbance indicated an increase in reducing power.

#### 3.6.2. DPPH Radical Scavenging Activity

The scavenging activity of 1,1-diphenyl-2-picrylhydrazyl (DPPH) radical was carried out according to Sachindra * et al.* [36]. Briefly, 2 mL 0.16 mM ethanolic DPPH solution was added to 2 mL of related solution. The mixture was shaken vigorously and left to stand for 30 min at room temperature in the dark, and then, the absorbance was measured at 517 nm. The inhibition of DPPH radicals by the samples was calculated as follows: DPPH radical scavenging activity (%) = [1 − (absorbance of sample − absorbance of blank)/absorbance of control)] × 100%.

#### 3.6.3. Hydroxyl Radical Scavenging Activity

Hydroxyl radical scavenging activity was determined based on Yang * et al.* [2] with minor modification. Briefly, 0.2 mL of chrysolaminarin CL2 solution were mixed with 2 mL EDTA-Fe solution (0.15 mM) and 0.8 mL salicylic acid (2 mM). Afterwards, 2 mL H_2_O_2_ (6 mM) were added to the reaction mixture and incubated for 30 min at 37 °C. Absorbance was measured at 510 nm. The capability of hydroxyl radical scavenging by the samples was calculated as follows: hydroxyl radical scavenging activity (%) = [1 − (absorbance of sample − absorbance of blank)/Abs. of control)] × 100%.

## 4. Conclusions

The new chrysolaminarin, CL2, was purified from marine diatom, *O. aurita*, and structurally determined as a glucan linked by the β-d-(1→3) (main chain) and β-d-(1→6) (side chain) glycosidic bond. The results of antioxidant experiments indicate that CL2 possesses potent antioxidant, especially for scavenging hydroxyl radicals. The high volumetric productivity of chrysolaminarin in the column photobioreactor suggests that the microalga *O. aurita* could be developed as a new natural source of antioxidant or as a food supplement.
